# Organic–Inorganic Combined Application: A Review of Long-Term Experimental Insights and Pathway to Sustainable Sugarcane Production

**DOI:** 10.3390/plants15203095

**Published:** 2026-10-10

**Authors:** B. R. Kulasekara, Jing Ai, Yutong Wang, Zhongfu Zhang, Shaolin Yang, Jingmei Dao, Jun Deng

**Affiliations:** 1 National Key Laboratory for Tropical Crop Breeding/Sugarcane Research Institute, Yunnan Academy of Agricultural Sciences, Kaiyuan 661699, China; kulasekara@sugarres.lk (B.R.K.); aijing@yaas.org.cn (J.A.); wangyt@yaas.org.cn (Y.W.); zhang@yaas.org.cn (Z.Z.); yangsl@yaas.org.cn (S.Y.); djm@yaas.org.cn (J.D.); 2 Sugarcane Research Institute, Uda Walawe 70190, Sri Lanka; 3 Yunnan Provincial Key Laboratory of Sugarcane Genetic Improvement, Kaiyuan 661699, China

**Keywords:** integrated nutrient management, nutrient use efficiency, organic–inorganic fertilization, soil health, soil microbiome, sugarcane industry by-products, sugarcane

## Abstract

This review synthesizes evidence from long-term field experiments, meta-analyses, and high-throughput sequencing studies on combined organic–inorganic fertilization, with a focus on sustainable sugarcane production in Yunnan, China. Prolonged use of inorganic fertilizers degrades soils through acidification, loss of organic matter, and reduced microbial diversity. Integrated nutrient management sustains crop productivity while maintaining soil health, outperforming inorganic-only applications. Organic amendments, including farmyard manure and sugarcane by-products such as pressmud, vinasse, and mill ash, improve soil structure, nutrient availability, and water retention while counteracting acidification. These amendments also reshape soil microbial communities, enriching beneficial taxa, suppressing pathogens, and increasing network stability, alongside functional shifts in N fixation, P and K solubilization, mycorrhizal symbioses, and plant growth promotion. Enhanced enzyme activities and functional microbial abundance drive improved C, N, and P cycling. Based on site-specific long-term trials, sugarcane industry by-products can replace 25–50% of inorganic fertilizer under conditions where environmental risks are appropriately managed. Future research should prioritize region-specific amendment strategies, precision agriculture tools, functional microbial characterization, standardized soil health indicators, and socioeconomic assessments of adoption constraints. This review provides a framework for transitioning to soil health-centric nutrient management, with implications for China’s sugar security and sustainable agriculture in Yunnan’s diverse landscapes and beyond.

## 1. Introduction

The Green Revolution (1950–1970) has been a major driver of the intensification of global agricultural production since the mid-20th century. Historically, this intensification has relied on the widespread use of chemical fertilizers to increase crop yields [1,2,3,4]. However, prolonged use of agrochemicals, particularly the exclusive use of inorganic fertilizers, has led to widespread soil degradation, including acidification, depletion of soil organic matter (SOM), loss of soil microbial biodiversity, and declining nutrient use efficiency (NUE) [1,2,3,4,5]. To address these adverse conditions, the combined application of organic and inorganic fertilizers has emerged as a cornerstone of integrated nutrient management (INM), offering a strategy to ensure crop productivity while maintaining long-term soil sustainability. This approach leverages the prompt, precise nutrient supply of inorganic fertilizers and the soil-conditioning, slow-release properties of organic amendments [4,6].

This INM framework is particularly relevant to high-biomass, intensively managed cash crops that require substantial nutrient inputs, with sugarcane (*Saccharum* spp. hybrids) representing the most important sweetener crop in China’s agricultural context. Sugar is a key agricultural commodity in China, essential to the national economy and a vital raw material for industries such as foods, beverages, and pharmaceuticals. The main types are cane sugar and beet sugar, with cane sugar accounting for nearly 80% of total production. It is primarily grown in the southern and southwestern provinces, including Guangxi, Yunnan, Guangdong, and Hainan. Guangxi is the leading producer, while Yunnan, the second-largest producer, is crucial to national sugar security. In Yunnan, over 70% of sugarcane is grown on dryland hills, where soils have low organic matter and poor structure. Implementing combined organic–inorganic fertilization with industrial by-products such as pressmud, vinasse, and mill ash can improve soil fertility, water retention, and yields. Developing region-specific INM approaches for Yunnan’s diverse, hilly landscapes is vital to safeguarding China’s sugar security.

**Figure 1:**
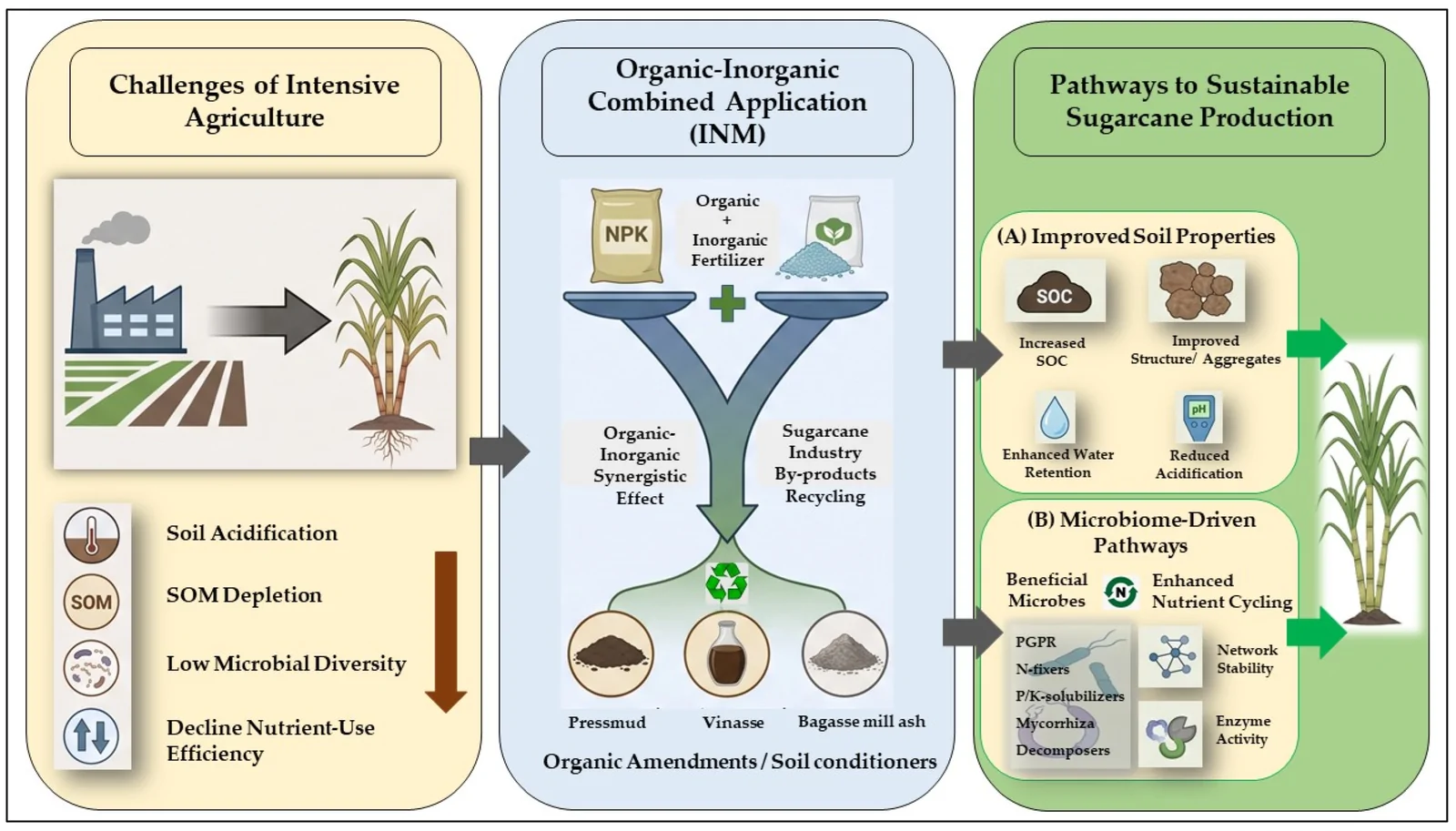
Conceptual diagram of organic–inorganic combined fertilization for sustainable sugarcane production. The diagram shows the progression from the challenges in intensive agriculture, through the INM solution that combines organic–inorganic approaches with sugarcane by-products as organic amendments/soil conditioners, to the resulting outcomes: improved soil properties and microbiome-driven pathways that collectively enhance nutrient cycling, soil health, and crop productivity.

With this context established, the present review synthesizes recent advances in research conducted in China and internationally on combined organic–inorganic fertilization in agriculture, with a focus on soil management in sugarcane cultivation. It evaluates the impacts of this practice on crop yield, soil physicochemical properties, and soil microbial communities, with particular attention paid to the synergistic mechanisms linking these factors. Given its high nutrient demand and extended ratoon-cropping cycle, commercial sugarcane cultivation is highlighted as a key crop for which integrated nutrient strategies hold significant promise for enhancing both productivity and environmental sustainability [5,6]. The principles of INM have been successfully applied at the commercial level to many perennial and high-value crops [6,7]. The integration of organic manures, farmyard manure, and industrial by-products with chemical fertilizers and biofertilizers, including *Azotobacter*, phosphate-solubilizing bacteria (PSB), and arbuscular mycorrhizal fungi (AMF), has been shown to improve soil health, reduce nutrient leaching, and enhance crop quality and yield [3,6,7]. These evidence-based insights provide a valuable pathway for developing INM strategies in sugarcane production systems (Figure 1).

Importantly, a rapidly emerging focus in research at both domestic and international levels is the development of robust frameworks for soil health assessment that extend beyond traditional physicochemical indicators [7,8]. This holistic approach encompasses soil chemical, physical, biological, and ecological metrics, including microbial community composition, enzyme activities, C mineralization potential, and soil food web structure. Holistic soil health assessment is a prerequisite for sustainable agriculture. This approach provides early warning signs of degradation, guides adaptive nutrient management decisions, and verifies the long-term efficacy of practices such as combined organic–inorganic application. Without such assessment, yield gains may obscure underlying declines in soil functionality. Therefore, future INM research and studies must integrate standardized soil health indices to evaluate not only crop yield but also the resilience and regenerative capacity of agroecosystems, making soil health assessment both a scientific and policy imperative for global food security [8,9].

## 2. Literature Search and Synthesis Methodology

This review synthesizes evidence from the peer-reviewed literature on combined organic–inorganic fertilization in agricultural systems. A comprehensive literature search was conducted across Web of Science Core Collection, Scopus, China National Knowledge Infrastructure (CNKI), Google Scholar, and PubMed. While foundational studies on organic–inorganic integration and soil health exist, studies from the 2010–2026 period were selected based on methodological advances, including high-throughput sequencing technologies, meta-analytical approaches, and detailed mechanistic understanding of soil–plant–microbe interactions. Therefore, this review builds upon that foundation while focusing on the recent methodologically advanced literature and recent policy guidelines emphasizing integrated nutrient management in China.

The literature search was performed during February to May 2026. Studies were screened based on their relevance to the review topic, and all retrieved studies were published in English-language peer-reviewed journals. Search terms included combinations of: organic–inorganic fertilization, integrated nutrient management, long-term experiment, soil health, microbial community, sugarcane, and sugarcane industry by-products. Studies were included if they reported the effects of combined organic–inorganic fertilization and provided relevant data; those not meeting these criteria were excluded. A small number of foundational studies (pre-2010) were included due to their unique and still-relevant insights into the utilization of sugarcane industry by-products as soil amendments.

Priority was given to long-term field experiments, meta-analyses, and studies employing high-throughput sequencing. Given that sugarcane is a perennial crop with an average 12-month growing period and multiple ratoon harvests, experiments lasting more than 3 years were considered long-term, while those spanning 2–3 years were considered medium-term, including trials across consecutive crop cycles. Shorter-term studies, including lysimeter and pot experiments, were included as supplementary evidence to support the primary findings. Data were extracted from 60 eligible studies spanning diverse agro-ecological zones in China, India, Pakistan, Brazil, the USA, and sub-Saharan Africa. Extracted variables included experimental design, yield parameters, soil physicochemical properties, microbial community characteristics, and organic amendment properties. Quantitative findings are presented as ranges, with mechanistic interpretations drawn from the original studies. The review draws on long-term sugarcane experiments conducted locally and internationally, supplemented by shorter-term studies to validate findings across contrasting agro-ecological contexts.

## 3. Current Status of Research on Organic–Inorganic Combined Application in Agriculture

The integration of organic and inorganic nutrient sources in agriculture has a long history. However, its scientific underpinnings and importance have been more widely discussed over the past three decades. Previous research mainly focused on yield responses, whereas contemporary research increasingly emphasizes the mechanistic pathways through which combined fertilization influences soil functioning and crop performance [5,7,10]. This shift reflects the growing recognition that sustainable intensification requires not merely substituting chemical fertilizer with organic material, but also understanding their interactions within the soil–plant continuum. Consequently, current investigations have moved beyond general agronomic comparisons to explore the synergistic effects of organic–inorganic combinations on soil physical structure, chemical fertility, the microbial community, and its dynamics [8,10].

Research over recent decades has consistently demonstrated the agronomic and soil health benefits of the combined approach. Among these contributions, information from long-term field experiments is invaluable for understanding slow-changing soil processes and has provided compelling evidence. For example, in a soybean-maize intercropping system in China, Lin et al. [10] found that a 1:1 mixture of chemical and organic N fertilizer increased maize starch content by 8.4–9.2% compared with chemical fertilizer alone. Similarly, a two-year sugarcane study in Nanning, Guangxi, China, showed that the combined application of controlled-release urea and organic fertilizer achieved the highest sugar yield (17.7 t ha^−1^) and enabled a 46.4% reduction in N application compared with conventional fertilization. Furthermore, the organic–inorganic combination enhanced microbial diversity, enriched beneficial taxa involved in C, N, and P cycling, and reduced fungal pathogens, thereby promoting sustainable sugarcane productivity [11]. In a three-year sugarcane study in India’s north-central plain zone, Kumar et al. [12] demonstrated that INM using bio-resources (pressmud, vermicompost, farmyard manure) alongside inorganic fertilizers significantly improved cane yield (95.5–96.4 t ha^−1^), juice quality (commercial cane sugar up to 12.40%), and economic returns (benefit–cost ratio up to 1.52) compared with conventional fertilization.

SOM comprises all organic compounds in soil, including living biomass, fresh residues, and humus. Soil organic C (SOC) is the C fraction of SOM, typically accounting for 50–58% of SOM by weight [8,13]. SOM denotes the total organic pool, whereas SOC refers exclusively to its C component. Building on this foundation, the positive effects of combined fertilization on crop yields have been documented across a wide range of crops and agro-ecological zones. Table 1 summarizes selected key field experiments evaluating the effects of combined organic–inorganic fertilization on sugarcane yield, soil properties, and microbial communities across different geographic locations.

Combining organic and inorganic fertilization typically leads to higher yields and better soil quality compared to using chemical fertilizers alone. In Indian sugarcane systems, INM has been extensively documented to increase yield, improve quality, and enhance NUE compared with conventional methods. Similarly, research in China, Brazil, and the USA provides strong evidence that combining inorganic fertilizers, organic manure, biofertilizers, sugarcane industry by-products, and eco-friendly trash management significantly boosts cane and sugar production and supports system sustainability. These benefits stem from a more balanced nutrient supply, improved soil physical conditions, and heightened biological activity, all of which promote nutrient cycling and absorption.

However, it is important to highlight that the benefits of organic–inorganic fertilization are determined by different factors, and this integration is not superior for every system. For example, in a seven-year field study in the Vertisols of Telangana, India, Reddy et al. [21] demonstrated that inorganic fertilization maximizes sugarcane yield, whereas organic farming improves juice quality but faces economic constraints due to higher input costs. This finding underscores that the performance of INM varies with soil, climate, amendment quality, and management practices. For instance, in sandy soils with low organic matter and low nutrient retention, organic matter amendments can improve the water-holding capacity and nutrient availability, while in calcareous or saline soils, they may counteract salinization and alkalization. Therefore, the success of organic substitution depends on site-specific and region-specific INM strategies to balance productivity and profitability.

The response to organic–inorganic integration varies with baseline soil fertility [22]. In a rice production system in Hunan, China, a 60% chemical fertilizer plus 40% organic fertilizer (pig manure, ryegrass, rice straw) produced the greatest increases in total N, P, and organic matter in medium- and high-fertility fields, whereas 100% chemical fertilizer was most effective in low-fertility fields [22]. Furthermore, soil enzyme activities (urease, acid phosphatase, sucrase) are consistently higher under organic–inorganic combinations, and the combined enzyme activity index is also higher in medium- and high-fertility fields [22]. These findings underscore the need to tailor fertilizer recommendations to baseline soil fertility to achieve optimal agronomic and economic returns.

The economic returns of INM are also significant. By substituting a proportion of chemical fertilizers with organic sources, farmers can reduce input costs while maintaining or increasing productivity, thereby improving overall profitability and operational sustainability [3,23]. In the context of sugarcane, integrated production systems that incorporate sugar-industry by-products, such as pressmud, mill ash, and concentrated vinasse, have demonstrated enhanced nutrient recycling. Furthermore, this integration provides profitable returns through reduced chemical fertilizer consumption and improved energy cogeneration [24].

Beyond sugarcane, the benefits of integrating organic and inorganic inputs have been extensively documented across diverse agricultural systems. A 20-year energy-based assessment of the agricultural ecosystem in Dazhou, China (2002–2022), found that increasing organic inputs alone did not enhance system sustainability [25]. Although organic energy inputs rise, industrial auxiliary inputs, particularly chemical fertilizers, remain the predominant component of the energy input structure. Concurrently, the energy yield ratio declined, the environmental load ratio increased substantially, and the lower energy sustainability index indicates a prolonged low level of sustainable development capacity [25]. These results suggest that incremental increases in organic fertilization are insufficient to offset the adverse effects of heavy reliance on industrial auxiliary inputs. Achieving meaningful improvements in sustainability requires a systemic approach that integrates agricultural practices to reduce dependence on chemical fertilizers, enhance resource-use efficiency, and promote ecological agricultural practices.

These summarized findings demonstrate that the principles of INM are broadly applicable across diverse agricultural systems. A study of drip-irrigated spring wheat in Xinjiang, China, found that replacing 23% of chemical N with organic fertilizer increased wheat yield by 3.85% and improved N use efficiency by 40.46% compared with a conventionally chemical-fertilized system [26]. Xiong and Zhao [27] identified a significant substitution relationship between fertilizer use efficiency and application quantity, finding that N fertilizer inputs have already exceeded the optimal threshold for grain production. This finding is critically important for informing policy-level changes in China aimed at reducing the overuse of chemical fertilizers in agriculture.

Furthermore, organic–inorganic integration improves the efficiency of soil nutrient cycling and reduces environmental nutrient losses. C-nutrient stoichiometry studies show that stable SOM storage requires N, P, and S to be stored with C in fixed proportions [28], indicating that supplementary nutrients are necessary to enhance C sequestration and support the INM approach. In Inner Mongolia, China, combined organic–inorganic fertilization of saline–alkali soils reduced salinity by 11.1–22.8% in the 0–60 cm soil layer, decreased pH from 8.50 to 8.45 by 0.05 units, and increased the redox potential of the surface layer by 2.5–4.3% [29]. These changes collectively improved soil conditions, thereby facilitating crop establishment. These multifaceted benefits underscore INM as a strategic pathway for sustainable intensification across heterogeneous soil types and cropping systems.

## 4. Current Research on the Impact of Organic–Inorganic Combined Application on Soil Physicochemical Properties and Microorganisms

The beneficial effects of integrated organic–inorganic fertilization on crop yield and quality arise from synergistic changes in soil physical, chemical, and biological properties. Therefore, studies have shifted from nutrient measurement to understanding the complex interactions underpinning the soil ecosystem.

### 4.1. Impact on Soil Physicochemical Properties

The addition of organic matter is the primary driver of changes in soil properties. Organic amendments such as farmyard manure, compost, and sugar-industry by-products act as binding agents, promoting the formation and stabilization of soil aggregates. This improved soil structure increases porosity, improves aeration, enhances moisture retention, increases water infiltration, and lowers bulk density [29,30,31]. For sandy soils, which are inherently poorly structured and have low nutrient-retention capacity, the effects of these physical improvements are particularly pronounced. For example, applying sugarcane pressmud to sandy soil can more than triple SOM content, substantially increasing the soil’s water-holding capacity and providing a more favorable environment for root development [31,32].

According to Yu et al. [29], replacing a portion of urea fertilizer with an organic amendment in saline–alkali farmland soils reduces soil salinity, lowers soil pH, and increases soil redox potential compared with conventional fertilization. The organic–inorganic integration reduces the accumulation of ammonium-N and nitrate-N in the soil while increasing the accumulation of dissolved organic C, organic N, microbial biomass C, and microbial biomass N [29].

The synergistic effects of combined organic amendments have also been demonstrated with biochar and animal wastes. In a two-year field study in Nigeria, Adekiya et al. [33] reported that the combined application of biochar (50 t ha^−1^) and poultry manure (5 t ha^−1^) significantly reduced soil bulk density, increased porosity and moisture content, and enhanced soil chemical properties, including pH, organic matter, N, P, K, Ca, and Mg, compared with biochar or manure applied alone [33]. In terms of soil chemistry, the organic–inorganic combined strategy increases SOC and provides a more balanced nutrient supply. Studies on sugarcane have also demonstrated that INM improves the availability of SOC, N, and P [11,14], while long-term straw management practices influence soil P dynamics [16,17,18].

One of the most critical chemical interactions is the buffering effect of organic matter against soil acidification. Continuous application of nitrogenous chemical fertilizers, especially urea, is a major cause of soil acidification [1,9,32]. To quantify this effect, Deng et al. [9] reported that each kilogram of urea applied to the soil decreases soil pH by 0.0004 to 0.0012 units. Furthermore, the study highlights that the optimal N rates are 150 kg ha^−1^ for wheat and 200 kg ha^−1^ for maize. Organic amendments, through their carbonates and functional groups that bind abundant hydrogen and Al ions in the soil, help counteract this process. Studies on Chinese croplands have demonstrated that long-term application of manure combined with inorganic fertilizer can mitigate soil acidification. In contrast, chemical fertilizer-only treatments lead to a significant decline in pH [1,9].

Beyond these physical and chemical benefits, soil health, defined as the continued capacity of soil to function as a living ecosystem, links soil management to broader sustainability goals, including C sequestration, water quality, and biodiversity conservation [8]. This holistic approach builds on the earlier concept of soil quality [13]. In recent sugarcane studies, 55 years of non-burning in Brazilian sugarcane increased SOC by 78%, total N by 48%, total S by 78%, and total P by 2.6–3.0% [18], and similarly, Xu et al. [20] reported that combining bagasse (170 t ha^−1^) with ammonium nitrate improved soil physical (bulk density, water-holding capacity) and chemical (CEC, organic matter, P, K) properties in Florida mineral soils. These findings confirm that integrated management is essential for nutrient availability, sustained productivity, and long-term agroecosystem resilience.

### 4.2. Impact on Soil Microorganisms

The microbial community in a soil ecosystem is the most dynamic and responsive component, and its structure and functional organization are profoundly shaped by fertilization practices [33]. Long-term use of chemical fertilizers alone reduces microbial diversity, simplifies community structure, and alters functional characteristics, often favoring a few dominant, fast-growing microbial species [34]. In contrast, incorporating organic amendments into the soil provides a continuous, diverse supply of C and energy for soil microbes, thereby fostering a more abundant, diverse, and functionally resilient microbial community [33,35].

High-throughput sequencing refers to modern DNA sequencing technologies used in soil microbial research, which have also revealed specific compositional shifts induced by organic–inorganic combinations. In a sugarcane study conducted in the calcareous soils of the Indo-Gangetic Plains, India, Sinha et al. [15] reported that the integrated application of organic manure, biofertilizers, and inorganic fertilizers significantly enhanced soil microbial populations and enzymatic activities compared with inorganic fertilizer alone. Actinobacteria are functionally important in soil for decomposing complex organic compounds such as cellulose and lignin and for producing antibiotics that can suppress plant pathogens [33,36].

Network stability refers to a more interconnected microbial community, which is organized for greater resilience to external environmental stress. In a sugarcane field in Yunnan, Deng et al. [32] reported that combining chemical and organic fertilizers selectively enriched specific microbial groups, producing a more modular network with a higher proportion of positive co-occurrence edges. Supporting these findings, Kumar et al. [36] observed that integrating organic manure, biofertilizers, and inorganic fertilizers significantly increased soil microbial populations and enzymatic activities (urease and phosphatase) compared with inorganic fertilizer alone in a sugarcane plant-ratoon system in the Indo-Gangetic Plains of India. Such network stability and diversity are associated with greater resilience to environmental stresses and more efficient resource use within the soil ecosystem.

The influence of INM also extends to key functional groups involved in nutrient cycling. In paddy soil, with green manure integration, Chinese milk vetch (*Astragalus sinicus* L.) combined with reduced inorganic NPK (60–80% of the recommended dose) significantly increased SOM and potential nitrification activity compared with chemical fertilizer alone [34]. Green manure increased ammonia-oxidizing bacteria (AOB) abundance but decreased ammonia-oxidizing archaea (AOA) abundance, shifting the AOA-to-AOB ratio from >6.0 under chemical fertilizer alone to <1.0 [34]. This shift from AOA- to AOB-dominated ammonia oxidation indicates that green manure integration enhances soil nitrification potential and moderates microbial community structure in the soil. These findings demonstrate that chemical N fertilizer can be partially replaced by green manure while enhancing soil fertility and improving N cycling efficiency.

Similar findings have been reported from a Mollisol in China, where long-term inorganic NPK fertilization significantly altered the communities of AOA and AOB, enriching an acidophilic Nitrosotalea species [37]. Adding manure to the inorganic NPK regime not only increased overall ammonia-oxidizer abundance but also reversed the community shifts caused by NPK alone, resulting in a composition more similar to that of the unfertilized control. This integrated approach also produced the highest potential nitrification activity, which was strongly correlated with changes in AOA and AOB community composition [37].

Metagenomic analysis has identified shifts in S cycling functions under organic fertilization. Li et al. [38] observed that adding organic amendments to sugarcane fields in Fujian, China, notably increased genes related to sulfate import (*cysP*, *cysV*, *cysW*) and the assimilatory sulfate reduction pathway (*cysNC*, *cysN*, *cysD*, *sat*, *cysC*, *cysH*), suggesting enhanced S assimilation and biomass conversion. These functional shifts, along with enrichment of phyla such as *Acidobacteria*, *Proteobacteria*, *Chloroflexi*, and *Gemmatimonadetes*, correlated with higher soil pH, organic matter, and sugarcane yield [38]. This emphasizes the importance of organic fertilizers in boosting S cycling, a vital yet often underappreciated nutrient pathway in sugarcane cultivation.

Beyond enriching beneficial taxa, organic and biofertilizers can actively suppress soilborne pathogens. Liu et al. [39] reported that applying a biofertilizer to sugarcane in Guangxi, China, significantly reduced the abundance of *Leifsonia*, the causal agent of ratoon stunting disease, while enriching beneficial bacteria such as *Acidobacteria* and *Chloroflexi*. Furthermore, co-occurrence network analysis revealed that biofertilizer-treated soils exhibited network characteristics resembling those of healthy soils, with reduced centralization and increased modularity [39].

Similarly, in a sugarcane pot experiment in Guangxi, China, Chen et al. [40] reported that a bio-organic fertilizer formulation containing compost, *Bacillus*, *Trichoderma*, biochar, and CaCO_3_ significantly increased bacterial diversity and altered both alpha- and beta-diversity. This treatment enriched beneficial genera such as *Pseudomonas* and *Meyerozyma* by 50% while suppressing *Rozellomycota*, a fungal phylum associated with disease, thereby enhancing sugarcane growth and suppressing smut disease [40]. These findings demonstrate that biofertilizers contribute to disease suppression and improved soil health by targeting the restructuring of the rhizosphere microbiome.

Similarly, a study in Thailand on sugarcane showed that a bio-organic amendment supplemented with phosphate-solubilizing bacteria (PSB) and arbuscular mycorrhizal fungi (AMF) significantly improved sugarcane cultivation. Juntahum et al. [41] reported that combining compost with AMF and PSB produced the highest AMF colonization (51%), increased soil enzymatic hydrolysis activity, and enhanced bacterial populations compared with individual treatments. Key AMF genera enriched included *Glomus*, *Acaulospora*, and *Gigaspora*, along with the bacterium *Pantoea dispersa* [41].

The combined use of organics and inorganics also affects soil enzymatic activities, which indicate microbial metabolic potential and nutrient cycling rates. Studies have reported that integrated fertilization in sugarcane soils significantly increases urease and acid phosphatase activities compared with chemical fertilizer alone [32]. These enzymatic changes are closely linked to shifts in microbial community composition and serve as sensitive indicators of soil health improvement under INM practices. Table 2 summarizes microbial community responses to combined organic–inorganic fertilization reported in selected sugarcane field studies.

### 4.3. Synergistic Effects on Soil Quality, Microbial Function, and Crop Productivity

While the effects of organic–inorganic combined fertilization on soil properties and microorganisms are well documented (Table 1 and Table 2), an increasing number of studies are focused on elucidating the complex synergistic interactions among soil physicochemical and microbial properties that influence crop growth. This holistic perspective moves beyond simple soil–plant correlations to explore the mechanistic pathways by which management practices influence the soil–plant–microbe continuum [5,14,32].

The application of organic amendments to the soil triggers a cascade of interrelated effects. The direct addition of organic matter improves soil structure, creating a more favorable habitat for soil biota and enhancing aeration and water retention [5,8,30]. This improved soil environment promotes a more extensive and healthier root system, capable of exploring a larger volume of soil for nutrients and water [30,31]. Simultaneously, the organic matter serves as a primary energy source for heterotrophic microorganisms in the soil, stimulating their growth and biochemical activities. This enhanced microbial activity drives the decomposition of the organic material, leading to the gradual release of N, P, and S in plant-available forms through mineralization [28]. Thus, physical improvement and biological activation of the soil are not independent events but are tightly coupled and interrelated.

Understanding this organic–inorganic synergistic effect requires examining the reciprocal interactions within the soil system. Enhanced root growth, resulting from improved soil structure, increases the volume and diversity of rhizodeposits, the organic compounds exuded by plant roots. These root exudates are rich in labile C, which further enhances microbial activity in the rhizosphere, the soil zone surrounding plant roots. This selective stimulation of soil environments enriches specific plant-growth-promoting rhizobacteria (PGPR), which produce phytohormones, enhance nutrient solubilization and mobility, or suppress soilborne pathogens, thereby benefiting plant health and growth [32,41]. According to Deng et al. [32], combined fertilization in sugarcane not only altered microbial community structure but also significantly increased the activity of soil enzymes, such as urease and acid phosphatase, which are direct products of microbial metabolism and are crucial for N and P cycling in the environment. These enzymes are crucial for converting organic nutrients into plant-available inorganic forms.

The C-nutrient stoichiometry of SOM is critical because it ensures balanced nutrient availability in the soil. Stable humus formation requires not only C but also N, P, and S in relatively fixed proportions. This implies that supplementary nutrients may be necessary to enhance C sequestration in agricultural soils [28].

Advanced statistical models help elucidate complex relationships among soil and crop factors. Structural equation modeling enables researchers to test hypothesized relationships among these soil and crop factors [32,35]. In a sugarcane system, Partial Least Squares Path Modeling analysis revealed that combined organic–inorganic fertilization did not directly affect sugarcane yield [32]. Instead, it influenced yield indirectly by modifying soil properties and enzyme activity, thereby shaping the microbial community structure. Therefore, these findings suggest that microbially mediated pathways contribute to final crop productivity [32,39].

In recent research, soil health assessment frameworks, such as the Soil Management Assessment Framework, have emerged as a prominent, high-priority area of interest. These frameworks have proven effective at evaluating synergistic effects by integrating physical, chemical, and biological indicators into a comprehensive soil quality index [42,43]. Studies of Brazilian sugarcane expansion using the Soil Management Assessment Framework revealed distinct differences in soil functional capacity. Soils under native vegetation functioned at 87% of their potential, whereas pasture soils functioned at 70% and sugarcane soils at 74%. Additionally, significant correlations have been identified between soil quality index (SQI) scores and SOC stocks [42]. Consistent with these findings, comparative evaluations of soil quality indexing strategies have shown that simple, user-friendly approaches are as effective as complex ones at detecting changes in soil quality.

Beyond statistically powered methods, field-based soil quality indexing offers a practical, low-cost alternative approach for soil quality assessment. According to Cherubin et al. [43], a comparison of six soil quality indexing strategies revealed that land-use change effects in Brazilian sugarcane systems could be sufficiently detected using a minimum dataset of five indicators. These indicators included pH, P, K, Visual Evaluation of Soil Structure (VESS) score, and SOC concentration, combined with proportional weighting to reflect chemical, physical, and biological processes. This finding has important practical implications for monitoring soil health and assessing the feasibility of sugarcane expansion areas. This approach enables cost-effective and rapid assessment without compromising scientific rigor. Collectively, these findings provide a mechanistic understanding of why organic–inorganic combined application is successful. The fundamental principle lies in managing the soil as an integrated, living system by fostering a healthy physicochemical environment that supports a beneficial, active microbiome.

## 5. Sugarcane By-Product Generation and Utilization for Sustainable Nutrient Management

Sugarcane is a long-duration cash crop cultivated through consecutive plant and ratoon cycles, with substantial nutrient requirements that necessitate the application of INM principles. An average sugarcane crop yielding 100 tons of cane per hectare removes approximately 208 kg of N, 53 kg of P, 280 kg of K, 30 kg of S, 3.4 kg of Fe, 1.2 kg of Mn, and 0.6 kg of Cu from the soil [44].

**Figure 2:**
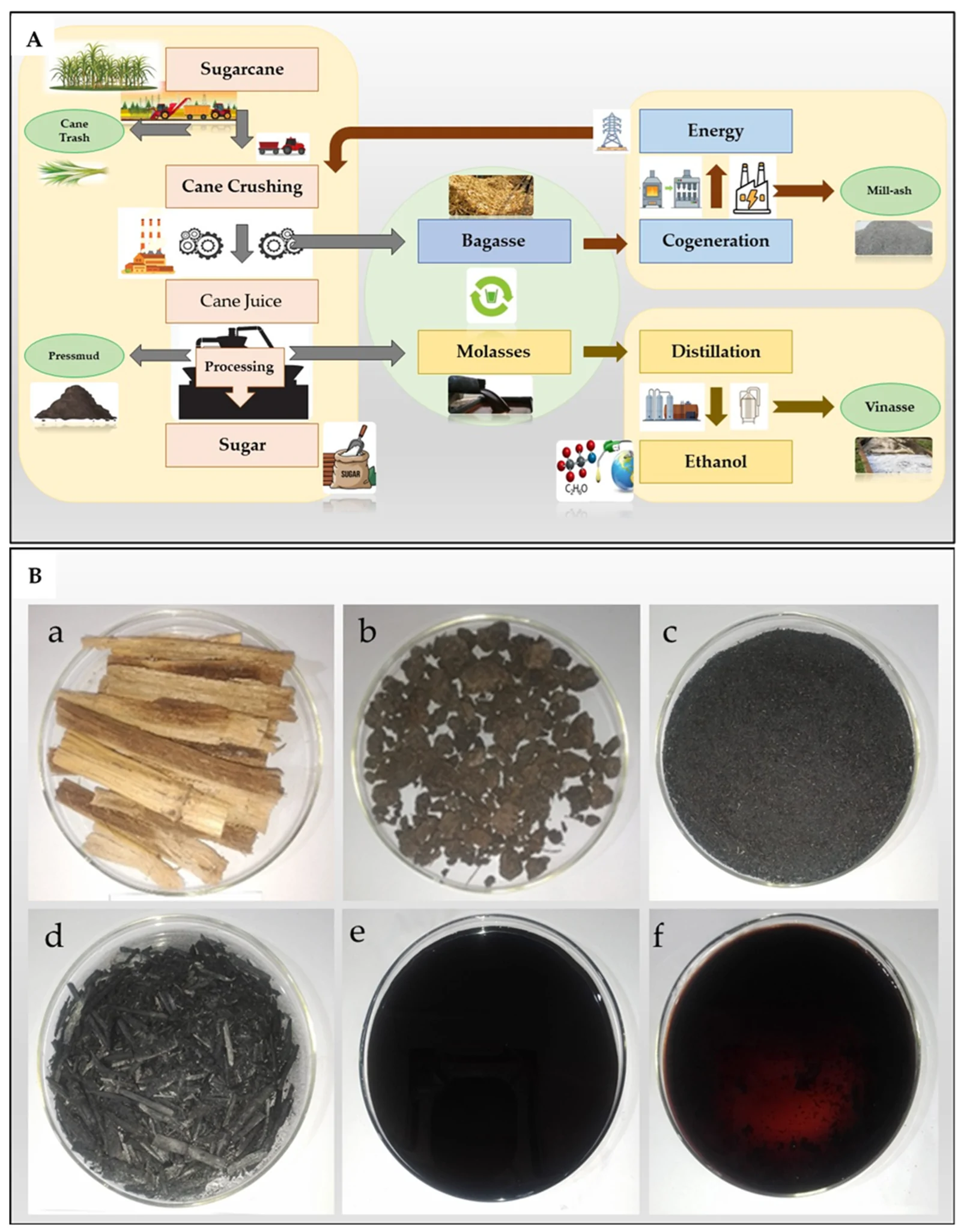
Schematic of the sugarcane processing chain and its by-product portfolio. (**A**) Industrial process diagram illustrating the production routes for sugar and ethanol, along with co-product streams and a self-sustaining cogeneration system that recycles energy back into the process. (**B**) Representative by-products and value-added products from the sugarcane industry: (**a**) bagasse, (**b**) pressmud (filter cake), (**c**) mill ash (boiler ash), (**d**) bagasse-biochar, (**e**) molasses, (**f**) vinasse (spent wash).

Sugarcane is the primary raw material for cane sugar production worldwide, accounting for 80% of global sugar output. The production process involves several sequential steps, including harvesting, sugarcane preparation, extraction, clarification, filtration, evaporation, crystallization, centrifugation, and drying. The sugarcane industry produces numerous by-products, and managing them is a significant environmental challenge. A typical sugar-alcohol industry generates bagasse, bagasse mill ash, molasses, pressmud, and vinasse as main by-products [45]. The industrial processing line, along with product development, utilization, and recycling pathways, is illustrated in Figure 2A, while representative by-product samples are shown in Figure 2B.

Sustainable handling of these by-products is important given their environmental impact, and substantial evidence supports their utilization to reduce inorganic fertilizer use in sugarcane [45]. Research on organic–inorganic combinations in sugarcane has a long history. It has intensified in recent years, driven by concerns about soil degradation from chemical fertilizers, declining fertilizer use efficiency, the need for a holistic approach to soil health, and the need to improve sugarcane performance.

### 5.1. Sugarcane By-Products as Organic Amendments

A significant amount of research has investigated the use of sugarcane industry by-products as organic amendments, representing a prime example of product recycling in action. Pressmud (filter cake/filter mud), a residue from juice clarification, and vinasse (spent wash), a liquid effluent from alcohol distillation, are produced in vast quantities. Globally, processing one ton of sugarcane generates approximately 30–35 kg of pressmud and 140–160 L of vinasse [45]. Their direct disposal into soil as sugarcane pressmud and vinasse presents environmental challenges, but studies have consistently demonstrated their value as fertilizers and soil conditioners.

Pressmud is a soft, spongy, amorphous material that ranges from dark brown to brownish and is rich in organic matter, P, and micronutrients. Its application improves soil fertility, increases cation exchange capacity, and enhances the availability of nutrients such as P and K [31,46]. In calcareous soils of the Indo-Gangetic Plains, Jha and Thakur [47] demonstrated that supplementing 100% of the recommended NPK fertilizers with pressmud, neem cake, and seaweed extract increased cane yield by 21.34% and sugar yield by 22.65% compared with the recommended dose of chemical fertilizers alone. The combined application of pressmud at reduced rates of recommended fertilizers produces cane and sugar yields equivalent to those from the full recommended doses of chemical fertilizers alone, resulting in significant savings in fertilizer costs [31]. Hence, this clearly demonstrates actual replacement of mineral fertilizer, distinguishing it from supplementation where organic amendments are applied in addition to recommended inorganic fertilizer rates. Furthermore, according to Gopalsundaram et al. [44], in India, applying 10 metric tons of pressmud per hectare, along with 75–100 kg of N fertilizer per hectare, increases N use efficiency by 4–8% and enhances SOM content and the availability of macro- and microelements.

Beyond raw pressmud application, composted and enriched pressmud, with the addition of rock phosphate, zinc sulfate, magnesium sulfate, gypsum, and microbial inoculants, further enhances its agronomic value. In a rice field study in India, a basal application of enriched pressmud compost at 1.25 t ha^−1^, supplemented with inorganic N as a top-dressing, produced grain yields comparable to those obtained with 2.50 t ha^−1^ of enriched pressmud compost alone [48]. This represents a 50% reduction in organic amendment rate when combined with inorganic fertilizer, demonstrating the synergistic effect of integrated nutrient management. Furthermore, the enriched pressmud also increased available soil N by 23.2%, P by 41.3%, and K by 22.3% compared with raw pressmud, while significantly increasing populations of soil bacteria, actinomycetes, and fungi [48]. These quantifiable improvements confirm that organic–inorganic integration not only reduces the required application rate but also enhances agronomic efficiency and crop productivity more effectively than organic or inorganic fertilization alone.

Vinasse is an aqueous by-product of ethanol production. It appears as a dark brown slurry with an unpleasant odor. It has a high potential for pollution due to its elevated organic load. The biochemical oxygen demand (BOD) of vinasse ranges from 20,000 to 40,000 mg/L, while its chemical oxygen demand (COD) ranges from 80,000 to 90,000 mg/L. In addition, vinasse is strongly acidic and corrosive. Importantly, vinasse, primarily a source of K (3100–5000 mg L^−1^) and water (90–93% moisture), has been widely used for diluted irrigation or as a direct fertigation [46,49]. Long-term studies show positive effects on soil chemical properties, particularly increased K, Ca, and organic matter, as well as on sugarcane productivity, provided it is applied at appropriate rates to avoid salinization and groundwater contamination [46,49]. These benefits are largely attributed to vinasse’s high organic load and nutrient content, which enhance soil fertility and microbial activity over time.

Similarly, a Brazilian study demonstrated that vinasse amendment effectively restored SOC quality after fire-induced disturbance in sugarcane fields, with vinasse-treated soils exhibiting the highest total organic C (65.06 g kg^−1^) and improved humic acid structures compared to burned soils without amendment [17].

Furthermore, integrating vinasse with other organic amendments, such as sugarcane pressmud, has been shown to enhance nutrient recycling, improve soil physicochemical properties, and reduce chemical fertilizer use [24]. Composting pressmud with distillery vinasse has also been recommended as a sustainable and cost-effective approach to producing organomineral fertilizers, thereby addressing both waste disposal and soil fertility management challenges [50,51]. Table 3 presents the physicochemical characteristics, recommended application rates, and agronomic benefits of major sugarcane industry by-products used as organic or organomineral fertilizers.

### 5.2. Composting of Pressmud and Distillery Vinasse

Composting sugarcane pressmud with distillery vinasse has been extensively studied and implemented in sugarcane-growing countries worldwide as a sustainable approach to producing nutrient-rich organic fertilizers. The process involves mixing pressmud with biomethanated vinasse, typically at a 1:2.5 ratio, and a microbial consortium containing cellulolytic and lignin-degrading species, including *Phanerochaete chrysosporium*, *Trichurus spiralis*, *Trichoderma* spp., *Azotobacter*, and *Pseudomonas* [52]. The mixture is formed into windrows and aerated with an aerotiller three times per week. Temperatures reach up to 70 °C within 7–10 days, helping eliminate pathogens and weed seeds [45,52]. After an 8–12-week composting cycle, the finished product contains approximately 1.27% total N, 1.61% total P, 1.42% total K, and 15.98% organic C, with a C:N ratio of 10:1, making it a high-quality organic fertilizer comparable to farmyard manure but with superior nutrient content [48,52]. According to Bernhardt and Notcutt, South African composting trials demonstrated that pressmud could be effectively composted over six weeks with manual turning every four days, resulting in 52% weight loss and a final C:N ratio of 17–19, which is optimal for soil application [52].

However, the quality and safety of compost depend heavily on the nature of the raw materials used. Compost produced from municipal solid waste or poultry litter can carry potentially toxic trace elements, organic pollutants, and antimicrobial resistance determinants, posing risks to human health and the environment if not properly managed [50,51]. These findings underscore that while composting sugarcane by-products is highly beneficial, careful selection of raw materials and process monitoring are essential to ensure the safety and quality of the final product [45,51].

### 5.3. Value-Added Products and Technological Innovations

The development of value-added products from sugarcane by-products represents a significant research advancement. For example, in a Brazilian study by Olivério et al. [23], the organomineral fertilizer “BIOFOM” was produced by concentrating vinasse to 30–45% total solids and mixing it with pressmud, mill ash, and mineral supplements to form a granular fertilizer that is easy to apply in practice. BIOFOM is a proprietary product name and a registered trademark for an organomineral biofertilizer developed by a private company in Brazil. According to these studies, BIOFOM can effectively substitute for conventional mineral fertilizers. Greenhouse trials have demonstrated comparable or superior performance in nutrient accumulation and plant growth, attributed to its organic matter content (400–700 g/kg), which facilitates gradual nutrient release and improves soil properties [23,24].

For example, in sugarcane studies across different continents, Ethiopian research found that combining 46 kg N ha^−1^ with 15 t ha^−1^ compost maximized yields, producing 315.6 t ha^−1^ of cane and 41.35 t ha^−1^ of sugar, with a benefit–cost ratio of 1.12 [55]. Similarly, Indonesian studies improved this method by optimizing composting. In one case, bagasse–manure (1:1) compost inoculated with *Trichoderma harzianum* increased stem weight (158.36 kg per plot) and yield (97.7 t ha^−1^) compared to conventional fertilization. Another blend, bagasse–pressmud–manure (1:1:1) with a fungal consortium, resulted in the highest sucrose content (31.32% pol), as per the pressmud’s P and micronutrient content [56]. Microbial consortia helped break down organic matter more quickly and improved compost quality, consistently boosting sugarcane growth compared with treatments without inoculation [56]. These findings underline that optimal fertilization combined with biological composting are effective strategies for sustainable sugarcane production.

### 5.4. Biochar and Sugarcane Mill Ash (Boiler Ash) Applications

There is sufficient evidence that using biochar and sugarcane mill ash as soil amendments is a promising approach to improving sugarcane productivity. In Florida, USA, applying sugarcane mill ash at 2% and rice hull biochar at 2% by weight significantly increased biomass and sucrose yields compared with unamended controls [19]. Furthermore, incorporating biochar and mill ash significantly increased soil silicon concentrations. The standard grower practice of incorporating 6% sugarcane mill ash with 2% rice hull biochar produced the greatest yield improvements [19]. Silicon leaf concentrations in the treated plots ranged from sufficient to high (0.78–2.18%), whereas unamended controls exhibited silicon deficiency. Leaf silicon concentrations in this range are attributed to improved tolerance to drought and abiotic stress in sugarcane, as silicon enhances photosynthetic efficiency, reduces transpiration rates, and strengthens cell wall rigidity under water-limited conditions [19].

According to Ferreira et al. [57], applying sugarcane mill ash to an Oxisol under organic management significantly improves soil chemical properties, including higher pH, increased levels of P, K, and Mg, and enhanced base saturation, compared with unamended controls. Applying mill ash at rates of 5–30 t ha^−1^ raises soil pH from 6.45 to 7.08–7.18. It also reduces exchangeable Al, thereby alleviating Al toxicity, a major constraint in highly weathered tropical soils [57]. These findings indicate that organic amendments, such as biochar and sugarcane mill ash, can effectively improve sugarcane productivity on acidic tropical soils.

Furthermore, Zafeer et al. [58] reviewed the production and applications of sugarcane bagasse-based biochar, highlighting its potential as a soil amendment to improve soil physicochemical properties. Biochar derived from sugarcane bagasse exhibits high C content, enhanced surface area and porosity, and a neutral-to-alkaline pH, which collectively improve soil fertility, water retention, and nutrient availability. The review also demonstrated that biochar application enhances microbial activity, increases cation exchange capacity, and effectively reduces heavy metal uptake by plants, making it a promising sustainable material for agricultural and environmental applications [58].

### 5.5. Rhizosphere Microbiome Restructuring with Sugarcane By-Products

The impact of organic–inorganic integrations on the sugarcane rhizosphere microbiome is an active and expanding area of research. The combined use of chemical fertilizers and organic amendments significantly increases soil microbial biomass and alters community structure. For example, Yang et al. [49] reported that in Chinese sugarcane fields in Guangxi, applying vinasse (120 t ha^−1^, diluted 1:5 with water) and pressmud (150 t ha^−1^) significantly increased populations of fungi, bacteria, and actinomycetes compared with inorganic fertilizer alone. Vinasse yields the highest actinomycete counts, whereas pressmud produces the greatest increases in fungal and bacterial populations in the soil. These changes are accompanied by significantly higher activities of key soil enzymes, including cellulase, phosphatase, and aminopeptidase, indicating enhanced mineralization of C, P, and N. Vinasse-treated soils also exhibit a slightly lower pH (5.51 vs. 5.70) and higher total C and N contents, resulting in a lower C:N ratio, which favors greater N availability [49]. Therefore, these findings further confirm that vinasse and pressmud can effectively substitute for chemical fertilizers while improving rhizosphere diversity as well as soil and sugarcane productivity.

### 5.6. Utilization of Sugarcane Industry By-Products in Other Crops

While most research on the use of sugarcane by-products focuses on sugarcane cultivation, studies on other crops offer valuable insights into their agronomic potential and productivity. For example, in calcareous soil in Pakistan, applying sugarcane mill ash at 0.1–10% by weight, along with basal inorganic N:P:K (120:90:60 kg ha^−1^), significantly increased wheat grain yield compared with chemical fertilizer alone [53]. The maximum grain yield of 5.2 t ha^−1^ was achieved with 2.0% sugarcane mill ash, representing a 62.5% increase over the unamended control, which yielded 3.2 t ha^−1^. This increase in yield was attributed to reduced bulk density, increased porosity, and enhanced availability of P, K, and micronutrients following the amendment with sugarcane mill ash [53].

Similarly, a study in Pakistan showed that applying sugarcane mill ash at rates of 3–250 t ha^−1^, along with inorganic NPK fertilizer, significantly increased wheat grain yield compared with NPK alone, with the optimal rate at 50 t ha^−1^ [54]. Sugarcane mill ash reduced soil bulk density from 1.34 to 1.27 g cm^−3^, increased total porosity from 51.0% to 52.1%, and enhanced soil pH from 8.2 to 8.9, exchangeable Ca + Mg from 94 to 284 mg kg^−1^, available P from 7.5 to 49 mg kg^−1^, and available K from 172 to 230 mg kg^−1^. These findings suggest that sugarcane mill ash has substantial potential as a soil amendment to improve crop productivity and sustainability across different cropping systems [54].

### 5.7. Environmental Concerns and Some Negative Effects of Sugarcane Industry By-Products

Despite their recognized agronomic benefits, sugarcane industry by-products pose significant environmental risks if not managed properly. Vinasse, in particular, has a high pollution potential due to its elevated organic load (BOD: 20,000–40,000 mg/L; COD: 80,000–90,000 mg/L) and acidity (pH 3.5–5.0) [45,49]. Improper disposal or excessive application can cause soil salinization and sodification from high K and Na levels [46,49], groundwater contamination from nitrate leaching [46], and greenhouse gas emissions during storage and field application [45]. Additionally, fresh vinasse contains high concentrations of organic acids that can cause phytotoxicity [51], while pressmud and mill ash may contain trace elements that accumulate in soils over time [53,54]. Similarly, while biochar is often considered a beneficial soil amendment, feedstock-related risks, including the presence of heavy metals and polycyclic aromatic hydrocarbons (PAHs), must be carefully evaluated to ensure safe and sustainable application [58].

To mitigate these risks, by-product application must follow the recommended rates (Table 3) and be properly composted or diluted before field application. Regular monitoring of soil electrical conductivity, pH, and heavy metal concentrations is essential to prevent long-term soil degradation [46,53]. Furthermore, careful selection of raw materials and process monitoring during composting are critical to ensuring the safety and quality of the final product [45,50,51]. Integrated approaches that combine appropriate application rates, pretreatment methods, and site-specific management practices are necessary to ensure the sustainable use of sugarcane industry by-products in agricultural systems.

## 6. Practical Strategies for Sustainable Nutrient Management in Sugarcane

Improving fertilizer use efficiency, particularly N use efficiency, remains a critical challenge in sugarcane production, with N use efficiency typically ranging from 30 to 40% under field conditions [44]. INM addresses this through several practical strategies, including slow-release N fertilizers (e.g., sulfur-coated urea, nano-urea), split N application (two to three splits within 45–90 days after planting), band placement instead of broadcasting, and foliar N application (2.0–2.5%) during soil moisture stress [44]. The combined application of sugarcane pressmud (10–20 t ha^−1^) with 75–100 kg N ha^−1^ increases N use efficiency by 4–8% and enhances SOM content and the availability of N, P, K, S, Zn, and Mn [44]. Similarly, Kumar et al. [36] reported that integrating inorganic fertilizers with vermicompost and biofertilizers (*Azotobacter* + PSB) improved agronomic N use efficiency by 75% compared with chemical fertilizer alone.

**Figure 3:**
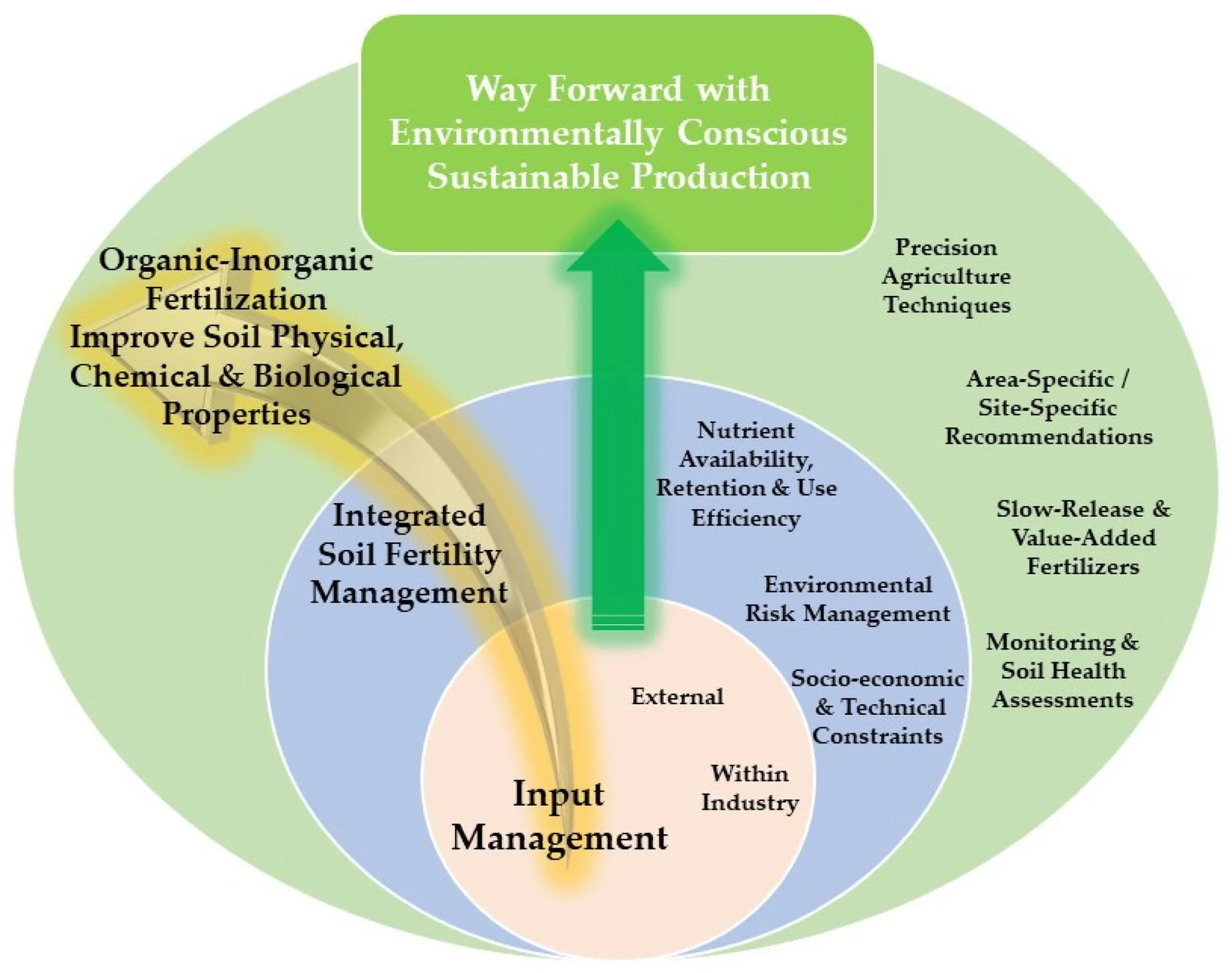
Conceptual framework for sustainable nutrient management in sugarcane, highlighting key strategies for improving soil fertility, nutrient use efficiency, and adoption of precision agriculture.

Beyond agronomic practices, sustainable sugarcane production requires INM principles adapted to local soil constraints and decision-making frameworks [59]. Region-specific strategies should integrate locally available organic amendments (e.g., pressmud, vinasse, mill ash) with inorganic fertilizers. Tailoring these applications to soil type and crop stage optimizes nutrient availability and minimizes environmental losses. International experiences confirm that integrated soil fertility management addresses soil acidification, secondary nutrient deficiencies, and soil physical constraints through the combined fertilization and improved germplasm [60]. Decision-support tools provide site-specific recommendations for smallholder farmers [59,60]. Local adaptation of fertilizer and organic input strategies to soil, climate, and socioeconomic conditions is critical for maximizing INM benefits. Therefore, future research should prioritize region-specific INM packages, precision agriculture tools, and long-term soil health assessments to support sustainable sugarcane intensification. Figure 3 illustrates key strategies for sustainable nutrient management, including organic–inorganic fertilization, precision agriculture, site-specific recommendations, and soil health monitoring.

## 7. Conclusions and Future Perspectives

This review synthesizes evidence on combined organic–inorganic fertilization in agricultural systems, with an emphasis on sugarcane production. Long-term trials demonstrate that INM can sustain higher yields compared with inorganic-only treatments under specific soil and climatic conditions. Yield benefits appear to be associated with microbiome-driven pathways, as combined inputs enrich beneficial soil microbiomes, increase network stability, and enhance C-N-P cycling. Structural equation modeling suggests that organic–inorganic combinations improve yield indirectly through microbially mediated pathways rather than through direct nutrient effects.

Based on site-specific long-term trials, sugarcane by-products, including pressmud, vinasse, bagasse, and mill ash, can replace 25–50% of inorganic fertilizer where environmental risks are managed appropriately. Composting pressmud with vinasse provides a sustainable approach to producing organomineral fertilizers, addressing both waste disposal and soil fertility challenges. However, careful management is essential to mitigate environmental risks, including soil salinization and groundwater contamination. Soil health assessment must incorporate biological indicators to verify the long-term sustainability of INM, as yield-centric evaluations may mask underlying declines in soil function.

Despite promising evidence, significant gaps remain in understanding the mechanisms underlying organic–inorganic interactions in sugarcane systems. Filling these gaps is crucial for optimizing INM strategies across diverse agroecological settings. Future research should focus on: regulatory mechanisms governing nutrient uptake at different growth stages, especially region- and variety-specific physiological and ecological responses; optimizing the ratios of sugar-processing by-products used as fertilizers and evaluating their long-term agronomic and environmental impacts; studying root system distribution and nutrient-use efficiency using advanced methods such as in situ root observation and isotope tracing; understanding how rhizosphere microbial communities influence yield through metagenomics and stable isotope techniques; and exploring interactions among different organic–inorganic fertilizers, including blended, compound, organomineral, and bio-organic types, and sugarcane varieties over planting cycles to guide precise fertilization and soil management. These mechanistic studies should be supported by region-specific trials of organic amendments, precision agriculture tools, microbial characterization, standardized soil health metrics, and socioeconomic evaluations of adoption barriers to ensure practical application.

By fostering a healthy physicochemical environment that supports a beneficial microbiome, this approach enhances nutrient cycling, improves soil health, and ensures long-term productivity, which is essential to safeguarding food security and building climate-resilient agricultural systems in Yunnan’s diverse landscapes and beyond. Therefore, adopting INM practices and prioritizing soil health are fundamental to securing the Chinese sugar industry and promoting sustainable agricultural development in ecologically diverse and economically significant sugarcane-growing regions of Yunnan, China.

## Figures and Tables

**Table 1:** Summary of field experiments evaluating the effects of organic–inorganic fertilization, crop residue management, and organic amendments on crop yields, soil properties, and microbial communities in sugarcane systems.

Location/Soil Type/Climate	Cropping System/Variety/Duration	Experimentation/Treatments	Key Findings	Reference
China—Nanning, Guangxi; red soil (Acrisols, WRB); Subtropical	Sugarcane Guitang-58 2 years (Plant + ratoon)	Conventional practice (560 kg N/ha); urea (300 kg N/ha); controlled-release urea (300 kg N/ha); 30% organic N substitution (300 kg N/ha); MCU: combined (urea + organic) (300 kg N/ha)	MCU—combined fertilization: highest sugar yield (17.7 t/ha); 46.4% N reduction; enhanced microbial diversity and C-N-P cycling; reduced fungal pathogens.	[11]
India—Lucknow; sandy loam alluvial (Inceptisols, USDA); semi-arid subtropical	Sugarcane 3 years (Plant + 2 ratoons)	T1: control (No N); T2: 100% inorganic N; T3: 90% inorganic + 10% FYM; T4: 80% inorganic + 20% FYM; T5: 70% inorganic + 30% FYM; T6: 60% inorganic + 40% FYM; T7: 50% inorganic + 50% FYM; T8: 100% FYM	Combined fertilization 50:50 (T7) reported the highest and sustained ratoon yield (66.57 t/ha), reduced soil bulk density (1.3 Mg/m^3^), improved soil infiltration, and increased SOC and microbial biomass.	[5]
India—Seorahi, Uttar Pradesh; soil with Medium OC, low P and K; sub-humid	Sugarcane (spring) Co S 09232 3 years (Plant + 2 ratoons)	T1: conventional practice; T2–T7: FYM/pressmud/vermicompost + 75–100% NPK + biofertilizers; T8-T9: Ghanjeevamrutha (BF) + Jeevamrutha (BF) + NPK; T10: natural farming	Best yield reported in combined fertilization: T6 (96.4 t/ha), T2 (95.5), T8 (95.2); 11–19% increase over control; best CCS: T4 (12.40%), T2 (12.37%); best benefit–cost ratio: T8 (1.52), T2 (1.50).	[12]
India—Bethuadahari, West Bengal; loamy soil; tropical	Sugarcane (spring) Co B 94164 3 years (Plant + 2 ratoons)	T1: control; T2: 100% recommended dose of inorganic—N (RDN); T3: 75% RDN + 25% FYM; T4: 50% RDN + 50% FYM; T5-T7: 75% RDN + 25% FYM + *Azotobacter*/*Azospirillum*	Best yield reported in combined fertilization T7: highest yield (86.1 t/ha), sucrose (17.52%); improved soil OC, N, P, K; 25% N substitution possible with FYM + biofertilizers.	[14]
India— Pusa, Bihar; calcareous/sandy loam (Entisol, USDA); subtropical	Sugarcane B.O.154 3 years (Plant + 2 ratoons)	T1: recommended dose of NPK; T2-T5: 100% NPK + *Acetobacter*/PSB/bio-compost; T6-T9: 75% NPK + *Acetobacter*/PSB/bio-compost	Best yield reported in combined fertilization T9: highest yield (85.8 t/ha), sugar yield (11.21 t/ha); reduced soil bulk density (1.32 Mg/m^3^); enhanced soil enzyme activities; possible to reduce 25% of inorganic NPK.	[15]
Brazil—Iracemapolis, Sao Paulo; clay soil (Oxisols, USDA, 2014); humid subtropical	Sugarcane IACSP-5000 6 years (Plant + 5 ratoons)	Straw (trash) maintenance (SM) experimentation: SM-15 (15 t ha^−1^); SM-10 (10 t ha^−1^); SM-5 (5 t ha^−1^); SM-0 (0 t ha^−1^)	Optimal straw retention (5 t ha^−1^) maintained sufficient labile P (25 mg kg^−1^) for sugarcane without P fertilizer; total removal caused P deficiency (12 mg kg^−1^); excessive retention (15 t ha^−1^) reduced organic P mineralization and plant availability.	[16]
Brazil—Campos dos Goytacazes, RJ; clay soil (Inceptisol, USDA, 2010); tropical savanna	Sugarcane 37-year burnt and 3-year unburnt crop comparison	SCB-37 (sugarcane burning 37 yrs); SCB-3 (sugarcane burning 3 yrs); SCV (sugarcane burning + vinasse 3 yrs); SCWB (sugarcane without burning 3 yrs)	SCV: highest TOC (65.06 g kg^−1^), SOC, POC, MOC, HA; fire reduced labile HA structures; ^13^C results showed vinasse application (SCV)—most effective strategy to reverse fire-induced SOM degradation.	[17]
Brazil—Campos dos Goytacazes, RJ; clay loam soil (Entisol, USDA, 2006); tropical savanna	Sugarcane 55 years (1946–2001)	Burned (pre-harvest burning) vs. unburned (no burning)	55 years of no burning have made soil organic matter more hydrophobic, which protects labile N, S, and P from rapid loss while keeping them available to crops.	[18]
USA—Belle Glade, Florida; sand soil (Arenic Alaquods, USDA)	Sugarcane-lysimeter study 2 years (Plant cane + ratoon)	Mill ash 6%, 2%, and 1%; rice hull biochar 2% and 1%; hardwood biochar 2% and 1%; horse manure + biochar 2%; horse manure + biochar 1%; control—fertilizer only.	Mill ash (2–6%) and rice hull biochar (2%) improved sugarcane yields over two cycles on sandy soils by supplying silicon and enhancing nutrient retention; rice hull biochar performed best among the biochars tested.	[19]
USA—Southern Florida; mineral soil (Entisol, USDA); tropical savanna	Sugarcane 4 years (Plant cane + ratoon)	Incorporation of bagasse B5—5 cm bagasse (85 t ha^−1^); B10—10 cm bagasse (170 t ha^−1^); B10 + N—10 cm bagasse (170 t ha^−1^) + 336 kg ha^−1^ ammonium nitrate; control—no bagasse	170 t ha^−1^ bagasse application improved soil health by reducing bulk density, increasing WHC, OM, nutrients, and microbial activity—making it a recommended amendment for Southern Florida sugarcane soils.	[20]

**Note:** BF, biofertilizer; FYM, farmyard manure; SCB, sugarcane burning; SCV, sugarcane with vinasse application; SCWB, sugarcane without burning; PSB, phosphate-solubilizing bacteria; OC, organic carbon; TOC, total organic carbon; POC, particulate organic carbon; MOC, mineral-associated organic carbon; HA, humic acid; SOM, soil organic matter; SOC, soil organic carbon; WHC, water holding capacity; USDA, United States Department of Agriculture; WRB, World Reference Base for Soil Resources; CCS, commercial cane sugar; 1000 kg = 1 metric ton (t).

**Table 2:** Microbial community responses to organic–inorganic combined fertilization in sugarcane systems.

Cropping System/Location	Fertilization/Treatments	Microbial Response	Key Organisms Enriched or Suppressed	Reference
Sugarcane (Guangxi, China)	Conventional practice; urea; controlled-release urea; 30% organic N substitution; combined (urea + organic)	Combined application increased bacterial diversity and network complexity, enhanced C-N-P cycling genes, and reduced fungal pathogens.	*Bacillus* and *Mortierella* enriched; pathotrophs reduced.	[11]
Sugarcane (Yunnan, China)	Control, F (chemical), FM (chemical + organic)	FM increased network complexity and positive edge proportion, and enhanced urease and acid phosphatase.	*Actinobacteria* correlated with yield (r = 0.590); beneficial taxa were enriched.	[32]
Sugarcane (Fujian, China)	F (chemical only), FM (chemical + organic)	OM enriched beneficial bacterial phyla and enhanced genes involved in metabolism and S cycling.	*Acidobacteria*, *Proteobacteria*, *Chloroflexi*, and *Gemmatimonadetes* enriched.	[38]
Sugarcane (Guangxi, China)	Urea, compound, BF1/BF2 (biofertilizer + urea)	Biofertilizer increased bacterial diversity, altered community composition, enhanced network stability, and reduced pathogenic bacteria.	*Acidobacteria* and *Chloroflexi* enriched; *Leifsonia* (pathogen of ratoon stunting disease (RSD) in sugarcane) suppressed.	[39]
Sugarcane pot experiment (Guangxi, China)	Control vs. BF (compost + Bacillus + *Trichoderma* + biochar + CaCO_3_)	BF increased bacterial diversity, altered alpha- and beta-diversity, enhanced crop growth, and suppressed sugarcane smut disease.	*Pseudomonas* and *Meyerozyma* enriched; *Rozellomycota* suppressed.	[40]
Sugarcane (Khon Kaen, Thailand)	Control, PSB, AMF, compost, mineral fertilizer, compost + AMF + PSB	Combined fertilization led to the highest AMF colonization, enzymatic hydrolysis, and bacterial populations.	*Glomus*, *Acaulospora*, and *Gigaspora* enriched; *Pantoea dispersa* suppressed.	[41]
Sugarcane (West Bengal, India)	Control, 100% RDN (inorganic), 75% RDN + 25% FYM, 50% RDN + 50% FYM, 75% RDN + 25% FYM + *Azotobacter*/*Azospirillum*	Combined fertilization increased microbial populations and improved soil health.	*Azotobacter*, *Azospirillum*, and PSB increased.	[14]

**Note:** F, chemical fertilizer; FM, chemical + organic fertilizer; BF, bio-organic fertilizer; PSB, phosphate-solubilizing bacteria; AMF, arbuscular mycorrhizal fungi; RDN, recommended dose of N; FYM, farmyard manure; OM, organic matter.

**Table 3:** Nutrient composition, application rates, and soil benefits of sugarcane industry by-products.

By-Product/Value Addition	OM (g kg^−1^)	N Content (g kg^−1^)	P Content (g kg^−1^)	K Content (g kg^−1^)	C:N Ratio	Recommended Rate	Key Benefits	Reference
Pressmud (Filter cake)	150–360	10–15	14–40	5–20	10:1	10–25 t ha^−1^	Increases soil CEC, P availability, and cane yield	[31,46]
Vinasse	70–90	0.41–0.56 (g L^−1^)	0.16–0.18 (g L^−1^)	3.1–5.0 (g L^−1^)	-	120–300 m^3^ ha^−1^ (diluted)	Source of K and water; increases soil K, Ca, and SOM	[46,49]
Enriched pressmud compost	200–280	13–19	15–20	14–17	10.6:1	1.25–2.50 t ha^−1^	Higher N, P, and K than raw pressmud; increases agronomic efficiency	[48,52]
BIOFOM (organomineral)	400–700	26–45	22–26	40–59	9:1	15–22 t ha^−1^	Gradual nutrient release; substitutes mineral fertilizers; recovers water from vinasse	[23,24]
Sugarcane mill ash	455	3	3	11	77:1	2–6% (*w*/*w*) or 50 t ha^−1^	Source of P, K, and micronutrients (Fe, Mn, Zn, Cu); improves soil physical properties.	[19,30,53,54]

**Note:** CEC, cation exchange capacity; SOM, soil organic matter; OM, organic matter; -, data not reported. OM and nutrient contents are expressed in g/kg on a dry-weight basis for solid amendments and in g/L for vinasse as per liquid amendment. 1000 kg = 1 metric ton (t).

## Data Availability

All data analyzed in this review are from previously published studies and are cited accordingly. No new primary data were generated during this study.
